# Impact of Pre-Gestational BMI and Gestational Weight Gain on Fetal Development Outcomes in Adolescent Pregnant Women

**DOI:** 10.3390/jcm13071839

**Published:** 2024-03-22

**Authors:** Orly Grobeisen-Duque, Oscar Villavicencio-Carrisoza, Carlos Daniel Mora-Vargas, Carolina Penelope Arteaga-Lopez, Maria Guadalupe Martinez-Salazar, Alejandro Rosas-Balan, Moises León-Juárez, Maria Isabel Villegas-Mota, Veronica Zaga-Clavellina, Ma. Guadalupe Aguilera-Arreola, Addy Cecilia Helguera-Repetto

**Affiliations:** 1 Departamento de Inmunobioquímica, Instituto Nacional de Perinatología Isidro Espinosa de los Reyes, Ciudad de Mexico 11000, Mexico; orly.grobeisen@gmail.com (O.G.-D.); cuauqbp@gmail.com (O.V.-C.); mora.vargas.daniel@hotmail.com (C.D.M.-V.); cari_tam@hotmail.com (C.P.A.-L.); salazarg63@yahoo.com (M.G.M.-S.); moisesleoninper@gmail.com (M.L.-J.); v.zagaclavellina@gmail.com (V.Z.-C.); 2 Escuela Nacional de Ciencias Biologicas del Instituto Politecnico Nacional, Ciudad de Mexico 11350, Mexico; marreoag@ipn.mx; 3 Instituto de Ciencias de la Salud, Universidad Autónoma del Estado de Hidalgo, Pachuca 42000, Mexico; 4 Coordinación de Medicina de la Adolescente, Instituto Nacional de Perinatología Isidro Espinosa de los Reyes, Ciudad de Mexico 11000, Mexico; alebalan1@yahoo.com.mx; 5 Unidad de Enfermedades Infecciosas y Epidemiología, Instituto Nacional de Perinatología Isidro Espinosa de los Reyes, Ciudad de Mexico 11000, Mexico; isavillegas13@outlook.com

**Keywords:** gestational weight gain, adolescent pregnancy, intrauterine growth restriction, birth weight, malnutrition, low body mass index

## Abstract

**Background.** Gestational weight gain (GWG) constitutes an essential aspect of the gestational process. Due to factors such as pregestational body mass index (BMI), nutritional intake, level of physical activity, and psychological aspects, the recommended GWG may not be achieved, leading to adverse neonatal outcomes. Adolescents, due to their physiological and mental developmental stage, are at a higher risk of inappropriate GWG. Our aim is to highlight the importance of GWG in our population and to determine the correlation with perinatal outcomes. **Methods.** Pregnant adolescents who attended a tertiary care institution for prenatal care were included; maternal data such as preBMI and GWG were used to determine maternal and neonatal outcomes using the chi-square test and OR determination. **Results.** A total of 202 adolescent pregnant patients were included, comprising those with inadequate GWG (*n* = 70), adequate GWG (*n* = 85), and excessive GWG (*n* = 47). A statistically significant association was found between low BMI and inadequate GWG. Patients with inadequate GWG demonstrated a correlation with IUGR and low birth weight, while patients with excessive GWG gave birth to macrosomic neonates. **Conclusion.** We concluded that previous habits play a significant role in determining weight gain throughout pregnancy. GWG has a direct impact on neonatal growth and development.

## 1. Introduction

Maternal weight gain during pregnancy is a well-established subject of study in the fields of nutrition and obstetrics. The effects of a balanced and proper diet not only impact the overall health of the mother but also significantly influence the growth and development of the fetus during gestation. The most recent guidelines from the American College of Obstetricians and Gynecologists (ACOG) recommend adhering to the Institute of Medicine (IOM) recommendations outlined in 2009 [1]. These guidelines emphasize that gestational weight gain (GWG) ought to be evaluated and managed according to the mother’s pre-gestational Body Mass Index (BMI) [2].

GWG constitutes an essential aspect of the gestational process. Throughout the establishment and progression of pregnancy, maternal weight experiences fluctuations due to various contributing factors. In the physiological and morphological domain, the development of the placenta adds approximately 0.5–1 kg to the mother’s weight, while the fetus itself averages 2.7–3.6 kg. Other factors, such as the formation of amniotic fluid (1 kg) and breast enlargement (0.5–1 kg), also contribute to weight gain during pregnancy [3].

Nonetheless, nutritional intake throughout the different trimesters is crucial. Understanding the changing nutritional requirements during pregnancy is essential for managing overall gestational weight gain. As a result, comprehensive prenatal care assumes a pivotal role in evaluating gestational weight gain and its repercussions on fetal health. Measures such as nutritional support and health education are fundamental for enhancing gestational health in both the mother and the fetus [4].

The social and psychological consequences of weight gain in adolescent patients can be a significant factor contributing to stigma. In 2017, Slof-Op ‘t Landt et al. conducted a study on the fear of weight gain among different ages and sexes, revealing a higher prevalence of this problem among women. The peak age for the fear of gaining weight was found to be between 16 and 25 years old, coinciding with higher rates of dieting. In adolescent pregnant women, this may affect gestational weight gain and consequently affect the overall health of both the mother and the fetus [5].

Inadequate weight gain during pregnancy has been associated with various adverse outcomes, including low birth weight (LBW), intrauterine growth restriction (IUGR), stillbirth, and preterm delivery, among others [6,7]. Therefore, it is crucial to raise awareness about the importance of achieving adequate weight gain and proper nutrition during pregnancy [4,8].

In Mexico, according to the latest statistics published by the ‘Instituto Nacional de Estadística y Geografía’ (INEGI; National Institute of Statistics and Geography), up to 15.1% of registered births were by adolescent mothers (less than 20 years old) [9]. The ‘Secretaría de Gobernación’ (Government Department) in Mexico reported that the adolescent fecundity rate was 60.3 per 1000 adolescents (aged 15–19 years old) in 2023 [10]. These data highlight a significant and often understudied problem that needs to be addressed. Adolescent pregnancy not only poses a significant challenge, but it also represents an ignored population within the health system. This demographic represents a high-risk group that is often overlooked. It is crucial to prioritize the prevention of adolescent pregnancy, rather than solely addressing the high prevalence and incidence of adolescent patients seeking appropriate healthcare. Consequently, there is a dearth of studies addressing the physiological development and healthcare recommendations for these adolescent patients during pregnancy, and the potential impact on both the mother and neonate’s health. There is no current information regarding recommendations for GWG during adolescent pregnancies.

In addition to the significant healthcare concern of adolescent pregnancy, we observe that this is a highly susceptible population, forced to navigate through pregnancy while also facing other common adolescent issues, such as social acceptance, peer pressure, substance abuse, mental health concerns, family dynamics, and body image, among others [11]. All of these factors profoundly impact the normal maturation of adolescents, contributing to a deficit in their overall biopsychosocial well-being.

In this study, our objective is to provide insight into the importance of gestational weight gain in adolescents pregnant patients treated at the “Instituto Nacional de Perinatología Isidro Espinosa de los Reyes”. Additionally, we aim to determine the relationship between inadequate weight gain during pregnancy and the occurrence of maternal and fetal/neonatal outcomes, such as gestational diabetes, preeclampsia, urinary tract infections, low birth weight, intrauterine growth restriction, and respiratory distress syndrome, among others.

## 2. Materials and Methods

### 2.1. Study Population

All participants provided their written informed assent, as they were adolescents. We included all pregnant adolescents who attended the “Instituto Nacional de Perinatologia “Isidro Espinosa de los Reyes” (INPer; National Institute of Perinatology) for prenatal care from January 2018 to December 2019 and met the inclusion criteria.

The inclusion criteria were as follows: pregnant adolescents aged 12–17 years old, gestational age before or equal to 19.6 weeks of gestation, consistent prenatal care leading to delivery at the Institute, and complete electronic medical records, including pregestational maternal weight. Exclusions encompassed patients over 17 years old, those with more than 19.6 weeks of gestation, and individuals with incomplete electronic records. Additionally, patients who withdrew their assent or did not receive prenatal care and delivery at the Institute were eliminated.

The inclusion criteria were designed to ensure optimal follow-up during visits by including patients with pregnancies up to 19.6 weeks. This criterion aimed to facilitate close monitoring of nutritional habits and psychological well-being. Pregnancies beyond the 20th week were excluded as they did not provide sufficient information for comprehensive pregnancy development and follow-up. As a result, the study focused on patients receiving comprehensive and specialized care from a multidisciplinary team, including healthcare providers such as nutritionists, psychologists, medical doctors, obstetricians, and maternal-fetal specialists.

### 2.2. GWG Identification and Groups Definition

Subsequently, we calculated GWG based on the guidelines of the Institute of Medicine and the recommendations of the American College of Obstetricians and Gynecologists (Table 1) [2]. We also explored associations between various maternal and neonatal outcomes. All data were obtained from the institutional electronic medical records. Maternal variables analyzed included pregestational BMI [12]. (categorized as underweight < 18.5, normal = 18.5–24.9, overweight = 25–29.9, and obesity > 30), urinary tract infections during pregnancy, substance abuse, premature rupture of membranes (PROM), chorioamnionitis, and preeclampsia. 

### 2.3. Fetal and Neonatal Outcomes Analysis

Additionally, we examined neonatal outcomes such as sepsis, meningitis, respiratory distress syndrome, tachypnea, intrauterine growth restriction, birth weight (categorized according to the World Health Organization [13,14] reference as low weight ≤ 2499 g, adequate 2500–3999 g, macrosomic ≥ 4000 g), and gestational age (late preterm 32–36.9 weeks of gestation, term 37–41.9 weeks of gestation, post-term ≥ 42 [14]. Neonatal sex was only used for descriptive purposes.

Throughout the article, we will refer to IUGR (Intrauterine Growth Restriction) as a fetus that had estimated weight gain calculated by ultrasound < 10th percentile, while the term Small for Gestational Age (SGA) will be used to express birth weight < 10th percentile corresponding to the gestational age (SGA-10p) or 2 standard deviation (SGA-2SD) folds to the left of the mean. Conversely, the term large for gestational age (LGA) will be used to refer to birth weight > 90th percentile corresponding to the gestational age, as macrosomia will be used to refer tp birth weight ≥ 4000 g [14,15].

### 2.4. Clinical Data Search and Statistical Analysis

Statistical analyses were performed using IBM SPSS Statistics 27 (SPSS Inc., Chicago, IL, USA). We obtained percentages and measures of central tendency, frequency calculation using One Way ANOVA for parametric variables; we also conducted comparisons using the chi-square test, with a significance level of *p* < 0.05. We assessed risk using odds ratio (OR) analysis based on 2 × 2 contingency tables, along with a 95% confidence interval (95% CI). Graphs were generated utilizing the ForestPloter 1.1.1 package on the R platform.

### 2.5. Ethical Considerations

This project received approval from the Ethics and Research Internal Review Board of the “Instituto Nacional de Perinatología” (INPer) in Mexico City under registration number 2017-3-131. Adhering to the ethical principles outlined in the Declaration of Helsinki, we ensured voluntary participation through informed assent and consent, maintained data anonymity, and upheld confidentiality. Given that our study involved adolescents, we obtained both signed assent from the participants and consent from their legally authorized representatives.

## 3. Results

A total of 685 adolescent pregnant patients sought medical care and delivered their babies at INPer during the period from January 2018 to December 2019. However, the analysis focused on a subset of 202 adolescent pregnant patients who met the study’s inclusion criteria. This smaller sample size was primarily due to incomplete data regarding pre-pregnancy weight and pre-conceptional visits starting after the 20th week of gestation. Consequently, this study selectively included patients who received comprehensive and meticulous care. The mean age was 15.82 ± 0.988 years (ranging from 13–17), and the mean gestational age was 38.48 ± 1.827 weeks (ranging from 32–43.5).

Following ACOG recommendations, we classified patients into three groups based on their GWG: inadequate GWG (*n* = 70), adequate GWG (*n* = 85), and excessive GWG (*n* = 47). The mean ages and gestational ages were similar among the three groups; the mean of maternal age was 15.82 ± 0.988 years (ranging from 13–17; *p* = 0.529 between the three groups), and the mean gestational age was 38.48 ± 1.827 weeks (ranging from 32–43.5). Analysis of pregestational BMI revealed an increase in low pregestational BMI among the inadequate GWG group (7.9%, compared to 3.0% and 2.5% in the adequate GWG and excessive GWG groups, respectively). Conversely, obesity was more prevalent in the excessive GWG group. Notably, premature rupture of membranes was more common in the inadequate GWG group (8.4%), while preeclampsia was more common in the patients with adequate GWG (4.5%) and excessive GWG (4%). The Apgar score was classified as either normal (≥7) or low (<7). Although we observed a higher incidence of low Apgar scores in the inadequate gestational weight gain (GWG) group, there was no statistically significant difference compared to the adequate GWG group (*p* = 0.224). Additionally, no cases with a score < 7 were found at 5 min for the Apgar scores. Preterm deliveries were more frequent in the inadequate GWG group, whereas full-term neonates were predominantly born to excessive GWG patients. The frequencies of maternal and neonatal characteristics and outcomes are summarized in Table 2.

**Figure 1:**
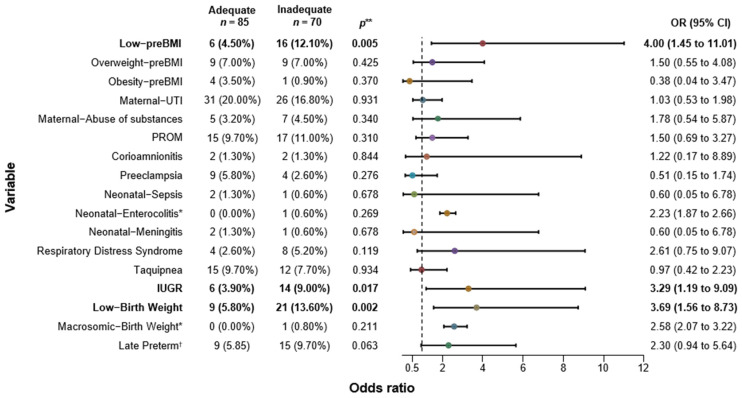
Association of Inadequate Gestational Weight Gain with Maternal and Neonatal Outcomes. preBMI (Pregestational Body Mass Index); UTI (Urinary Tract Infection); PROM (Premature Rupture of Membranes); IUGR (Intrauterine Growth Restriction); 95% CI (95% Confidence Interval; inferior and superior limits are shown); OR (Odds Ratio). * Relative Risk (RR) was calculated as OR was not possible to estimate. ** *p* ≤ 0.05. † 32–36.9 weeks of gestation; we did not find very preterm or extremely preterm in our population.

**Figure 2:**
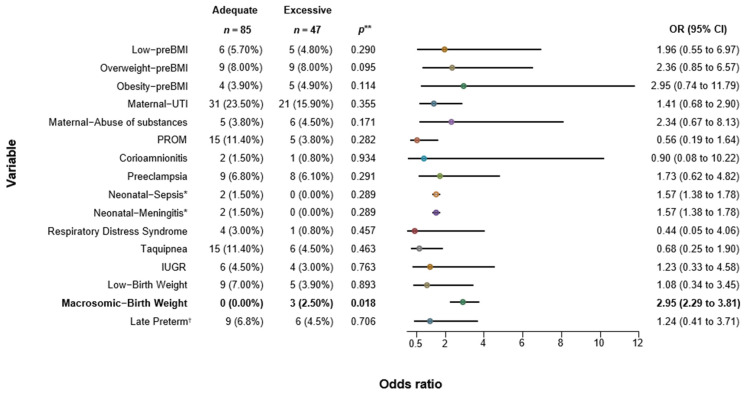
Association of Excessive Gestational Weight Gain with Maternal and Neonatal Outcomes. preBMI (Pregestational Body Mass Index); UTI (Urinary Tract Infection); PROM (Premature Rupture of Membranes); IUGR (Intrauterine Growth Restriction); 95% CI (95% Confidence Interval; inferior and superior limits are shown); OR (Odds Ratio). * Relative Risk (RR) calculated as OR was not possible to estimate. ** *p* < 0.05. † 32–36.9 weeks of gestation; we did not find very preterm or extremely preterm in our population.

We explored the association between GWG and maternal/neonatal outcomes. For inadequate GWG, we found a strong positive association with low pregestational BMI (*p* = 0.005), demonstrating that a low pre-BMI increased the risk of inadequate GWG during pregnancy by 4 folds (OR 4; CI: 1.45–11.01). No significant effect was observed for maternal outcomes, but for neonatal outcomes, we found that mothers with inadequate GWG had an increased risk of delivering newborns with IUGR (OR 3.29; CI 1.19–9.09) and/or low birth weight (OR 3.69; CI 1.56–8.73). Both outcomes were statistically significant (*p* = 0.017 and *p* = 0.002, respectively). Preterm births were also more common in inadequate GWG mothers, with an OR of 2.30; however, the confidence intervals crossed the null, rendering it not statistically significant (Figure 1). Figure 2 provides an analysis of mothers with excessive GWG. Pregestational BMI was not associated with excessive GWG, although there was a slight, non-significant trend observed for overweight women before pregnancy (*p* = 0.095). The only significant risk identified was the delivery of macrosomic neonates, with a risk increase of 2.95 times compared to adequate GWG mothers (OR 2.95; CI 2.28–3.80). The OR graph within the tables illustrates the graphical representation of whether the confidence interval of each analyzed variable crosses 1, indicating a quantitative association with inadequate WG (Figure 1) or excessive WG (Figure 2). In both instances, enterocolitis appears to demonstrate a positive association with inadequate or excessive WG. However, due to the probability being higher than 0.05, this effect is influenced by the occurrence in only one neonate. A similar situation is observed with sepsis and excessive WG.

We could not find a correlation between pre-gestational BMI, gestational weight gain (GWG), and diabetes. Among the cases of diabetes mellitus type 1 and 2 (DM1 and DM2) and gestational diabetes (DMG) in our population, we only encountered two instances. The first case was DM1, and the second was DMG. However, the association did not reach statistical significance. Moreover, it is noteworthy that the patient with DM1 had a low pregestational BMI and inadequate GWG, whereas the patient with DMG had a normal pregestational BMI and adequate GWG.

## 4. Discussion

During adolescence, women undergo hormonal and physiological changes, serving as a transitional period in the reproductive stage. Adolescent pregnant women, in addition to the changes already mentioned, also experience the typical pregnancy adaptation changes required for fetal development. Due to the elevated stress levels and specific physiological demands, these pregnancies are categorized as high risk.

Women of reproductive age, particularly during adolescence, should adhere to a comprehensive and balanced diet to fulfill their growth goals and ensure both physical and cognitive maturation. Pregnancy further amplifies the necessity for increased nutritional intake to adequately support fetal development [16]. However, due to their high-risk and unstable status, adolescent pregnant patients often struggle to meet these nutritional needs.

In our study of 202 adolescent pregnant patients, we found that more than half of them had inadequate or excessive GWG, representing as abnormal GWG. These results indicate a pathological pattern of weight gain within our population.

Our research demonstrated a significant correlation between low pregestational BMI and inadequate GWG (OR 4; CI 95% 1.45–11.01). These might be associated with various behavioral and psychological patterns observed in some of the analyzed cases, such as anorexia nervosa, bulimia nervosa, major depressive disorder, generalized anxiety disorder, depressive and anxious personality traits, low caloric intake, malnutrition, undernutrition, and efforts to prevent weight gain. Some adolescent pregnant patients concealed their pregnancies due to fear of family reprisal, resorting to dietary measures to prevent the visible signs of pregnancy.

These findings align with a study by Darling et al., focusing on adult women in low- and medium-income countries, showing that underweight pregestational women were at higher risk of inadequate and severely inadequate GWG [17]. This finding was associated with chronic undernutrition patterns since childhood, which affected their eating habits. The same authors also showed that women with overweight or obese pre-pregnancy BMIs had a lower risk of inadequate or severely inadequate GWG but a higher risk of excessive GWG compared to those with a normal BMI [17].

In another study, Yaw Yong et al. analyzed the correlation between pregestational BMI, height and weight with GWG rates and found that patients with pregestational underweight and normal weight had a higher prevalence of inadequate GWG during the third trimester of Malaysian adult women treated in urban clinics. Meanwhile, patients with overweight had a higher rate of excessive GWG during the third trimester [18].

Additionally, another study conducted on Malaysian pregnant women (aged 19–40 years old) associated high rates of inadequate GWG with underweight pregestational BMI, with a stronger correlation observed in undernourished patients, as categorized by mid-upper arm circumference (MUAC). Many studies have also suggested the use of MUAC as a more reliable predictor for future inadequate GWG, with a higher positive predictive value compared to the BMI [17,19], opening the possibility for using new tools to bring better and higher quality of healthcare to pregnant patients.

In a study conducted in France, Amyx et al. demonstrated that women with underweight pregestational BMI had a higher prevalence of inadequate GWG (OR 1.4 CI 1.2–1.5). Contrary to our results, the same authors found that patients with obese pregestational BMI tended to develop inadequate GWG (OR 1.5; CI 95% 1.4–1.7) [20]. They report that 15% of pregnant women are minors aged 25 or younger, but the proportion of adolescents is unknown.

An important association was noted among the French population with inadequate GWG and insufficient care (OR 1.12; CI 95% 1.1–1.4). Criteria defining insufficient care included incomplete late pregnancy insurance declaration within the first three months of pregnancy, inadequate sonographic measurements in the first trimester, non-attendance at prenatal visits, or an insufficient number of recommended sonograms based on French guidelines for low-risk women [20]. In contrast, the adolescent pregnant patients in our study received comprehensive care, with close monitoring by obstetricians, hematologists, psychiatrists, maternal-fetal medical doctors, nutritionists, and other healthcare professionals to ensure holistic care, promoting the wellness of both, mother and fetus. Even though there is a notable prevalence of adolescents exhibiting low pre-BMI and insufficient weight gain during pregnancy indicative of insufficient clinical care for our adolescent population, we have perceived that many aspects of pregnant adolescents’ health are covered. It is important to mention that we are analyzing this particular population independently from adult pregnant women who may have other factors contributing to their lack of gestational weight gain.

Power et al. studied patterns observed in non-Hispanic white rural populations in the United States (average age: 26 years old at delivery). They revealed that one-third of the population with underweight pregestational BMI had inadequate GWG, and nearly half of the total population gained less than the recommended weight, particularly during their first trimester [21]. In our study, we identified a pattern of inadequate GWG throughout the entire pregnancy. Those who followed the recommendations provided by the nutritionist achieved GWG within the recommended parameters, allowing for a healthy pregnancy without complications.

Hasan et al. found an inverse association between an increase in BMI before third-trimester and inadequate weight gain (OR 0.96; CI 95% 0.93–0.99) [22]. Although this supports our statement that underweight BMI is associated with inadequate GWG, this study did not account for pregestational BMI due to the lack of optimal pregnancy diagnosis, pregnancy data collection, and follow-up. In our study, patients were monitored from the early stages of pregnancy, which included a complete pregestational medical history.

Through our study, we found that adolescent pregnant women with inadequate gestational weight gain (GWG) delivered more neonates with a lower Apgar score at 1 min (<7) compared to patients with adequate and excessive GWG; even though this was not statistically different. Liu et al. conducted a study involving more than 9 million mothers and infants in the United States to explore associations between gestational weight gain and adverse birth outcomes. Their results indicate increased odds of low Apgar scores in babies born to mothers with inadequate GWG, particularly in women who in their pre-pregnancy had overweight or obesity grade 1 [23]. In another study conducted on the Tanzanian population, the results demonstrated that inadequate dietary intake in pregnant patients significantly increased the risk of causing low Apgar scores (2.5-fold) [24].

While our study did not uncover a distinct risk for a low Apgar score, it did reveal a notable trend that aligns with findings from another research project. The importance of this trend at one-minute lies in assessing the infant’s ability to withstand the birthing process [25]. Moreover, at five minutes, it serves as an indicator of the infant’s adjustment to extrauterine life. These findings prompt further investigation to determine whether gestational weight gain directly impacts the adaptation process in our particular population.

In our study, the impact of inadequate GWG was directly related to fetal growth rate, showing a higher tendency towards intrauterine growth restriction (OR 3.29; CI 95% 1.19–9.09). Another significant outcome was low birth weight (OR 3.69; CI 95% 1.56–8.73). The type of intrauterine growth restriction (IUGR) found in the fetuses was asymmetrical, which was associated with inadequate nutritional intake by the mother, leading to decreased growth and intrauterine development of the fetus. If the fetus does not experience proper growth during the gestational period, the newborn’s weight will be directly associated with smaller height and weight [26].

Conversely, our results demonstrated a direct correlation between mothers with excessive GWG and birth weight of their babies categorized as macrosomic (OR 2.95; CI 95% 2.28–3.81). Patients who experienced excessive weight gain exhibited a high caloric intake, particularly with carbohydrates. This increase in nutrient and resources delivered to the fetus contributed to an increased fetal size, thereby impacting the newborn’s weight [27].

Perumal et al. studied a Tanzanian population, where 51% had an inadequate GWG. Compared to the adequate GWG group, the severely inadequate GWG population had higher risk of IUGR (OR 1.27; CI 95% 1.06–1.52) and LBW (OR 1.64; CI 95% 1.24–2.16), along with the inadequate GWG (LBW-OR 1.17; CI 95% 0.87–1.57/IURG-OR 1.2; CI 95% 1.0–1.43). Contrarily, the population with excessive GWG (18%), had a higher prevalence of macrosomia (OR 1.52; CI 95% 1.0–2.31), showing the same pattern found in our study (Figure 1) [28].

In a study conducted in Bangladesh, using LBW-term (OR 1.8; CI 95% 1.3–2.5), SGA-10th (OR 1.4; CI 95% 1.1–1.8), and SGA 2SD (OR 1.8; CI 95% 1.3–2.4), the authors correlated inadequate GWG with a disruption in normal growth and fetal development [29]. Even though they did not use the ultrasonographic measures to determine IUGR, they associated the GWG with a smaller and underweight baby due to a marginally nourished population.

Studies in Japan and Taiwan found that insufficient gestational weight gain increased the risks of SGA and LBW [17,18]. Similarly, research in Brazil reaffirmed this trend, emphasizing the global impact of maternal weight gain on neonatal health [19].

A study by Dude et al. based on data collected from the Nulliparous Pregnancy Outcomes Study: Monitoring Mothers-To-Be (NuMoM2b), which included 8628 pregnant women, showed that patients with inadequate GWG had higher risk of SGA (OR 1.64; CI 95% 1.37–1.96). Inversely, patients with excessive GWG had a higher prevalence of LGA (OR 1.49; OR 95% 1.23–1.80). Among the studied population, 208 patients were adolescents, with 53 having inadequate GWG, 58 having adequate GWG, and 97 having excessive GWG [12]. Compared to our population, these adolescent pregnant patients show a different kind of alimentary disorder deviation, given that our patient had greater proportion of inadequate GWG and NuMoM2b, which showed a pattern of higher prevalence of excessive GWG among adolescent pregnant patients [30].

The impact of excessive GWG also affects maternal health. There is a direct association between excessive GWG and pathologies such as gestational diabetes, type 2 diabetes mellitus, and hypertensive disorders including gestational hypertension, preeclampsia, and HELLP syndrome [31]. However, in our study, this relationship was not statistically significant. These results may be attributed to the young age of the patients, the rigorous follow-up provided by the nutrition, maternofetal medicine, endocrinology, and cardiovascular departments, in addition to the absence of previous comorbidities.

In the modern era, adolescent women and adolescent pregnant patients face a significant biopsychosocial challenge: the stigma associated with weight gain. Slof-Op ‘t Landt et al. revealed that the peak age for fear of gaining weight was found in ages between 16–25 years old, which associated with greater likelihood of dieting [5]. Influenced by societal pressures, young women put their lives at risk in pursuit of unattainable beauty standards, leading to unhealthy habits that may result in serious eating disorders and higher mortality rates. This, coupled with their pregnant status, poses a danger not only to themselves but also to the adequate growth and development of their fetus.

Our article significantly contributes to the existing literature and knowledge in the realm of GWG during adolescent pregnancies, providing crucial insights into the interplay between GWG and neonatal outcomes. Unlike many studies that predominantly focus on adult populations, our research focuses on a high-risk and often neglected demographic. By investigating this population, we unveil a complex pattern of abnormal and pathological GWG, with more than half of the subjects exhibiting inadequate or excessive GWG. The correlation identified between low pregestational BMI and inadequate weight gain highlights various behavioral and psychosocial factors contributing to the phenomenon, ranging from eating disorders to efforts to conceal pregnancies. Moreover, our study distinguishes instead by analyzing the specific healthcare environment for adolescent pregnant patients, revealing a notable prevalence of inadequate GWG despite comprehensive care.

Furthermore, our findings add nuance to the existing literature by delving into the unique challenges faced by adolescent pregnant patients, such as societal pressures, peer dynamics, and mental health concerns. This comprehensive exploration of the biopsychosocial challenges encountered by the population enriches our understanding of the factors influencing GWG and maternal–fetal outcomes. This study not only highlights the prevalence of inadequate and excessive GWG with its associated risks but also underscores the need for tailored interventions and preventing strategies considering the influence of socio- cultural factors on adolescent pregnant. Thereby, our research fills a crucial gap in the literature and provides a foundation for targeted healthcare approaches and future investigations in the field of adolescent maternal health.

Strengths: Most studies typically overlook vulnerable populations; however, this research stands out as one of the pioneering investigations globally, specifically exploring the correlation between adolescent pregnancy and gestational weight gain. At the ‘Instituto Nacional de Perinatología’, patients attending the adolescent clinic benefit from comprehensive care engaging various departments, including cardiology, endocrinology, nutrition, psychology, psychiatry, maternofetal medicine, obstetrics, pediatrics, and neonatology. This collaborative approach ensures that patients with additional risks receive thorough guidance for optimal health outcomes. Despite these strengths, external factors such as social influences and the home environment pose challenges, making it difficult to guarantee strict adherence to medical, nutritional and psychological plans.

Study Limitations: One of the primary limitations of this research lies in its sample size. While the sample is relatively homogeneous, a more significant number of included patients could enhance our statistical power. Another constraint is our inability to enroll patients from the inception of their pregnancies, which could have allowed for a more substantial impact on ensuring proper nutrition and gestational weight gain. Unfortunately, as previously mentioned, our patients typically present at our institute during the late first trimester or early second trimester, often due to concealing their pregnancies initially. Additionally, our adolescent population exhibits several distinctive characteristics: they originate from marginalized communities, possess low incomes, encounter social discrimination, and may experience psychological disorders. These factors potentially introduce bias into the study, as the demographic makeup of our sample may not accurately reflect that of the broader adolescent population. Hence, it is crucial to underscore that studies on adolescent pregnancy should encompass participants from diverse socioeconomic backgrounds, offering varying opportunities to enrich our understanding of its implications.

## 5. Conclusions

In conclusion, our study offers valuable insights into the importance of gestational weight gain (GWG) among adolescent pregnant patients under care at the “Instituto Nacional de Perinatología Isidro de los Reyes”. The findings underscore the urgent requirement for raising awareness and implementing targeted interventions to uphold optimal maternal health. Our results highlight the prevalence of irregular GWG patterns within this specific demographic, emphasizing the potential risks linked with both inadequate and excessive GWG.

The implications of our research extend beyond statistical correlations, highlighting the unique challenges faced by adolescent mothers, including the societal stigma and the adoption of unhealthy nutritional measures. Recognizing the impact of early and continuous monitoring, our study advocates for a comprehensive, interdisciplinary approach to a perinatal care, integrating mental health support and nutritional counseling. 

Furthermore, this study not only addressed the primary objective of providing insights into the importance of GWG but has also successfully determined the significant relationship between pathological GWG during pregnancy and a spectrum of critical maternal and fetal/neonatal outcomes. The analysis elucidates the significant link between inadequate GWG with LBW and IUGR, and excessive GWG with macrosomia. These neonatal outcomes exert further complications in the developmental trajectory of the neonate and infant, potentially leading to additional challenges in their overall well-being. The insights gained from this study contribute valuable knowledge to the field, serving as a foundation for tailored strategies aimed to optimize the gestational outcomes and overall well-being of adolescent pregnant patients.

Balanced nutrition is critical for the proper development of both the mother and fetus during pregnancy. In the case of adolescent pregnant patients, the stigma surrounding weight gain creates a risk factor for inadequate weight gain, which can lead to a precarious environment during the gestational period and adverse outcomes such as low birth weight and intrauterine growth restriction.

Establishing an organized and well-communicated chain within the health system can significantly improve the early delivery of services. This is particularly important during pregnancy, when optimal, timely, and well-coordinated health care can have a positive impact on the health of both the mother and the fetus, thereby preventing potential adverse outcomes. Investing in prevention strategies improves health and reduces the financial burden of treating comorbidities and complications in both the mother and the child. Overall, preventing adolescent pregnancy is essential for promoting the health, well-being, and opportunities of young people and breaking the cycle of poverty and disadvantage. It also contributes to promoting gender equity and fostering better biopsychosocial development.

Previous habits significantly influence weight gain during pregnancy. Adopting unhealthy nutritional practices, such as restricting calories, carbohydrates, and fats without proper guidance, can endanger the health of both the mother and the developing fetus. A comprehensive healthcare approach is vital to promote holistic well-being, ensuring favorable outcomes during and after pregnancy. This approach creates a positive relationship with one’s body and weight, fostering a supportive environment for adolescent mothers to nurture their children safely and healthily.

Recommendations: Further research on the topic should be conducted with a more significant population to create a greater pool with greater statistically significant results, supporting the data described in this article. It is imperative to strengthen public health policies aimed at preventing adolescent pregnancy, particularly in regions facing substantial challenges in this regard.

## Figures and Tables

**Table 1:** Recommended Pregnancy Weight Gain According to Pregestational BMI.

Pre-Pregnancy Weight Category	Pre-Pregnancy Body Mass Index (kg/m^2^)	Recommended Range of Total Weight (lb)	Recommended Rates of Weight Gain in the Second and Third Trimester (lb) (Mean Range [lb/wk])
Underweight	Less than 18.5	28–40	1 (1–1.3)
Normal Weight	18.5–24.9	25–35	1 (0.8–1)
Overweight	25–29.9	15–25	0.6 (0.5–0.7)
Obese	30 and greater	11–20	0.5 (0.4–0.6)

* Calculations assume a 1.1–4.4 lb weight gain in the first trimester [2].

**Table 2:** Overview of Demographic and Clinical Profiles in Mothers and Neonates by Gestational Weight Gain.

Characteristics			Maternal GWG *n* = 202		
			Inadequate *n* = 70 Mean ± SD or No. (%)	Adequate *n* = 85 Mean ± SD or No. (%)	Excessive *n* = 47 Mean ± SD or No. (%)
	Maternal Age		15.71 ± 0.95	15.83 ± 1.05	15.89 ± 0.98
Maternal	Pregestational BMI	Low	16 (7.9%)	6 (3.0%)	5 (2.5%)
		Normal	44 (21.8%)	66 (32.7%)	28 (13.9%)
		Overweight	9 (4.5%)	9 (4.5%)	9 (4.5%)
		Obesity	1 (0.5%)	4 (2.0%)	5 (2.5%)
	Urinary Tract Infection		26 (12.9%)	31 (15.3%)	21 (10.4%)
	Substances Abuse		7 (3.5%)	5 (2.5%)	6 (3.0%)
	Premature Rupture of Membranes		17 (8.4%)	15 (7.4%)	5 (2.5%)
	Chorioamnionitis		2 (1.0%)	2 (1.0%)	1 (0.5%)
	Preeclampsia		4 (2.0%)	9 (4.5%)	8 (4.0%)
	DM1		1 (1.4%)	0	0
	GDM		0	1 (1.1%)	0
Neonatal	Sex	Feminine	42 (20.8%)	44 (21.8%)	12 (5.9%)
		Masculine	28 (13.9%)	41 (20.3%)	35 (17.3%)
	Apgar < 7	1 min	9 (12.8%)	6 (7.0%)	3 (6.3%)
		5 min	0	0	0
	Sepsis		1 (0.5%)	2 (1.0%)	0 (0.0%)
	Enterocolitis		1 (0.5%)	0 (0.0%)	0 (0.0%)
	Meningitis		1 (0.5%)	2 (1.0%)	0 (0.0%)
	Respiratory Distress Syndrome		8 (4.0%)	4 (2.0%)	1 (0.5%)
	Tachypnea		12 (5.9%)	15 (7.4%)	6 (3.0%)
	Intrauterine Growth Restriction		14 (6.9%)	6 (3.0%)	4 (2.0%)
	Weight	Low	21 (10.4%)	9 (4.5%)	5 (2.5%)
		Adequate	48 (23.8%)	76 (37.6%)	39 (19.3%)
		Macrosomic	1 (0.5%)	0 (0.0%)	3 (1.5%)
	Late Preterm (32–36.9 Weeks of Gestation) †		15 (7.4%)	6 (3.0%)	9 (4.5%)
	Full—Term (>37 Weeks of Gestation)		55 (27.2%)	41 (20.3%)	76 (37.6%)

GWG (Gestational Weight Gain); BMI (Body Mass Index). DM (Diabetes Mellitus); GDM (Gestational Diabetes Mellitus); † We did not find very preterm or extremely preterm in our population.

## Data Availability

The original contributions presented in the study are included in the article, further inquiries can be directed to the corresponding author.
